# Computer-Aided Medical Microbiology Monitoring Tool: A Strategy to Adapt to the SARS-CoV-2 Epidemic and That Highlights RT-PCR Consistency

**DOI:** 10.3389/fcimb.2021.594577

**Published:** 2021-09-13

**Authors:** Linda Mueller, Valentin Scherz, Gilbert Greub, Katia Jaton, Onya Opota

**Affiliations:** ^1^Insitute of Microbiology, Lausanne University Hospital and University of Lausanne, Lausanne, Switzerland; ^2^Infectious Diseases Service, Lausanne University Hospital and University of Lausanne, Lausanne, Switzerland

**Keywords:** COVID-19, SARS-CoV-2, RT-PCR, biomedical validation, R-script, algorithm, quality-monitoring, delta checks

## Abstract

Since the beginning of the COVID-19 pandemic, important health and regulatory decisions relied on SARS-CoV-2 reverse transcription polymerase chain reaction (RT-PCR) results. Our diagnostic laboratory faced a rapid increase in the number of SARS-CoV-2 RT-PCR. To maintain a rapid turnaround time, we moved from a case-by-case validation of RT-PCR results to an automated validation and immediate results transmission to clinicians. A quality-monitoring tool based on a homemade algorithm coded in R was developed, to preserve high quality and to track aberrant results. We present the results of this quality-monitoring tool applied to 35,137 RT-PCR results. Patients tested several times led to 4,939 pairwise comparisons: 88% concordant and 12% discrepant. The algorithm automatically solved 428 out of 573 discrepancies. The most likely explanation for these 573 discrepancies was related for 44.9% of the situations to the clinical evolution of the disease, 27.9% to preanalytical factors, and 25.3% to stochasticity of the assay. Finally, 11 discrepant results could not be explained, including 8 for which clinical data was not available. For patients repeatedly tested on the same day, the second result confirmed a first negative or positive result in 99.2% or 88.9% of cases, respectively. The implemented quality-monitoring strategy allowed to: i) assist the investigation of discrepant results ii) focus the attention of medical microbiologists onto results requiring a specific expertise and iii) maintain an acceptable turnaround time. This work highlights the high RT-PCR consistency for the detection of SARS-CoV-2 and the necessity for automated processes to handle a huge number of microbiological results while preserving quality.

## Introduction

The rapid spread of the COVID-19 pandemic caused unprecedented challenges for diagnostic microbiology laboratories. Rapid and high throughput SARS-CoV-2 reverse transcription polymerase chain reaction (RT-PCR) developed early during the crisis became the cornerstone of patient diagnosis as well as hospital and public health management (Caruana et al., 2020; Corman et al., 2020; Tadini et al., 2020). Consequently, microbiology laboratories were reorganized to respond to the high demand for SARS-CoV-2 testing (Posteraro et al., 2020). This situation required (i) the rapid adaptation of infrastructures, (ii) quick validation and implementation of new RT-PCR assays, (iii) working hour extension and new workforce employment. Yet, the quality of results provided by clinical microbiology laboratories, SARS-CoV-2 testing and routine analyzes, had to be maintained throughout the crisis.

Our molecular diagnostic laboratory located in a tertiary care university hospital faced a rapid increase in the number of SARS-CoV-2 PCR with up to 1,007 tests per days at the peak of the epidemic. Our analysis platform set was progressively extended from a high-throughput MDx platform, to the cobas 6800 system (introduced on 24.03.2020) and the Xpert Xpress SARS-CoV-2 assay (introduced on 21.04.2020) in response to the high volume of SARS-CoV-2 testing. Additionally, validation procedures had to be simplified in our laboratory. To ensure the best quality, two validation steps are usually applied prior to result transmission to clinicians: the technical validation of the assay followed by the biomedical validation of the results by medical microbiologists, who consider the specific clinical setting (Greub et al., 2015). Biomedical validation appeared as a bottleneck in the SARS-CoV-2 analytical workflow which could extend the turnaround time (TAT), with the risk of affecting clinical outcomes, infection prevention strategies and public health decisions (Hawkins, 2007). To maintain a minimal TAT, results were released to the clinicians after technical validation based on the FastFinder software (UgenTec NV, Hasselt, Belgium) that automatically analyzes RT-PCR amplification curves.

The limited experience in these newly implemented RT-PCR assays, including their performance (Kokkinakis et al., 2020), raised the need for an active surveillance of the quality of provided results. Delta checks are commonly used in clinical chemistry laboratories to monitor analytical throughputs that outreach capacity for sample-by-sample validation. “Delta checks” describes a process where discrepancies in sequential results of the same patient are detected to prompt repetition of the analysis (Schifman et al., 2017). We wondered whether a similar approach could be used to monitor the quality of SARS-CoV-2 results obtained in our laboratory. Thus, we developed a quality-monitoring methodology based on a homemade algorithm programmed in R to monitor SARS-CoV-2 RT-PCR results. Such methodology leveraged repeated testing to identify potential preanalytical or analytical culprits as well as cases requiring further biomedical investigations. The algorithm developed in-house aimed to restrict the list of discrepancies truly requiring investigation.

In this article, we present the results obtained through the application of our quality surveillance on data from the first four months of the COVID-19 crisis in our laboratory. Besides its role as a quality management tool, application of this surveillance allowed us to quickly gain knowledge about RT-PCR assays applied to a novel virus and new disease. In particular, this process allowed us to identify clinical specimen with significant added value (i.e. patients with unexpected discrepant results) and the presence of long-term carrying patients.

## Materials and Methods

### RT-PCR and Samples

Samples collected from patients with suspected COVID-19 or for screening were tested by RT-PCR, using either our high-throughput MDx platform (Greub et al., 2015), the cobas SARS-CoV-2 qualitative test (Roche, Basel, Switzerland) and the Xpert SARS-CoV-2 test (Cepheid, California, USA). The E gene was targeted by the RT-PCR performed on the MDx platform (Greub et al., 2015), as described by Corman and colleagues (Corman et al., 2020). The cobas SARS-CoV-2 targeted the E gene as well as the ORF1a/b and was performed according to the manufacturer guidelines. Finally, the Xpert SARS-CoV-2 test targeted both the N and the E gene. The three methods displayed similar performances for the detection of SARS-CoV-2 from various clinical specimens and similar cycle threshold (Ct) value when positive (Lieberman et al., 2020; Moran et al., 2020; Opota et al., 2020; Poljak et al., 2020). Samples were mainly collected from the upper respiratory tract. However, other types of samples were also tested and are listed in **Supplementary Table S1**. Data collection and analysis

Data was collected during the first four months of the epidemic in Switzerland (12.02.2020-12.06.2020) and included all SARS-CoV-2 RT-PCR analyzes conducted at the Institute of Microbiology of the Lausanne University Hospital (CHUV). Samples were collected from symptomatic as well as asymptomatic patients. However, at the beginning of the pandemic only symptomatic patients were tested for SARS-CoV-2. From 25.04.2020, the screening strategy was extended to all patients admitted at our hospital, including the asymptomatics (Moraz et al., 2020). SARS-CoV-2 RT-PCR results and basic contextual information were extracted from our Laboratory Information System (LIS) (MOLIS, CGM) and analyzed with R (Team, RC 2019) (version 3.6.1) language helped by packages from the *Tidyverse* (Wickham et al., 2019) environment.

### Discrepant Cases Identification and Classification

A R script was developed to automatically identify and classify discrepant cases. In this script, all analyzes from patients with multiple samples were compared to their previous results in a pairwise approach. Sample comparisons were then categorized as concordant or discrepant. Only discrepant results were further processed. These discrepancies (positive versus negative or conversely) between consecutive samples were classified based on i) Ct values, ii) samples types and iii) reception dates (**Supplementary Figure S1**). Based on these records, discrepancies were classified by the algorithm as described in **Supplementary Table S2**. The script was designed to compare each sample only to the last relevant result. As described in **Supplementary Table S2** (Patient A), a positive nasopharyngeal swab followed by a negative PCR in blood, and then later by another positive nasopharyngeal swab, will lead to only one discrepancy: the negative blood classified as a “Low yield” sample. The second nasopharyngeal swab will be classified as concordant with the previous nasopharyngeal swab. Of note, two nasopharyngeal samples taken more than 10 days apart once negative and once positive with a Ct > 35, would be classified as “Stochastic” (Patients B and D) and not as “Time delay” (as it is for Patient C). Indeed, when none of these criteria are met (Sample type is not a “Low Yield”, Ct are <35 and Delta Time between samples is <10 days) the result is classified as “To be investigated” (Patient E). Of note, discrepancies were classified according to the first matching criteria in the following order: “Low yield”, “Stochastic”, “Time delay” and “To be investigated”. Furthermore, a result from a patient with three samples or more can be involved in a concordant and a discrepant pairwise comparisons. Indeed, the second of his analyzes could be in agreement with the first result but discrepant with the third. This decisional algorithm is graphically represented in **Supplementary Figure S1**. Code of this algorithm is available on https://github.com/valscherz/SARS-CoV-2_discrepant_screen.

Discrepant samples classified as “To be investigated” by the algorithm were then manually investigated, classified and assigned a putative explanation for the observed discrepancy (**Supplementary Table S3**). In this manual analysis, a discrepancy between two nasopharyngeal swabs taken within the same period (< 24h) and collected in different units or different hospitals (compatible with differences in sampling quality) were imputed to “Sample quality”. When sampling sources were different (i.e. comparing an upper respiratory tract sample with a rectal swab), discrepancies were imputed to “Different sample types”. Discrepancies between samples collected less than 10 days apart but with indications in clinical records supporting a recent infection or recent recovery were classified as “Clinical context”. Finally, discrepancies compatible with none of these putative explanations were classified as “Unsolved”. For visualization purposes, classified discrepancies were grouped into corresponding testing phases or context: clinical context, preanalytical or stochastic (**Supplementary Tables S3** and **S4**).

RT-PCR analyses were not repeated on the discrepant samples.

## Results

### Post-Analytic Surveillance of SARS-CoV-2 RT-PCR Results

**Figure 1:**
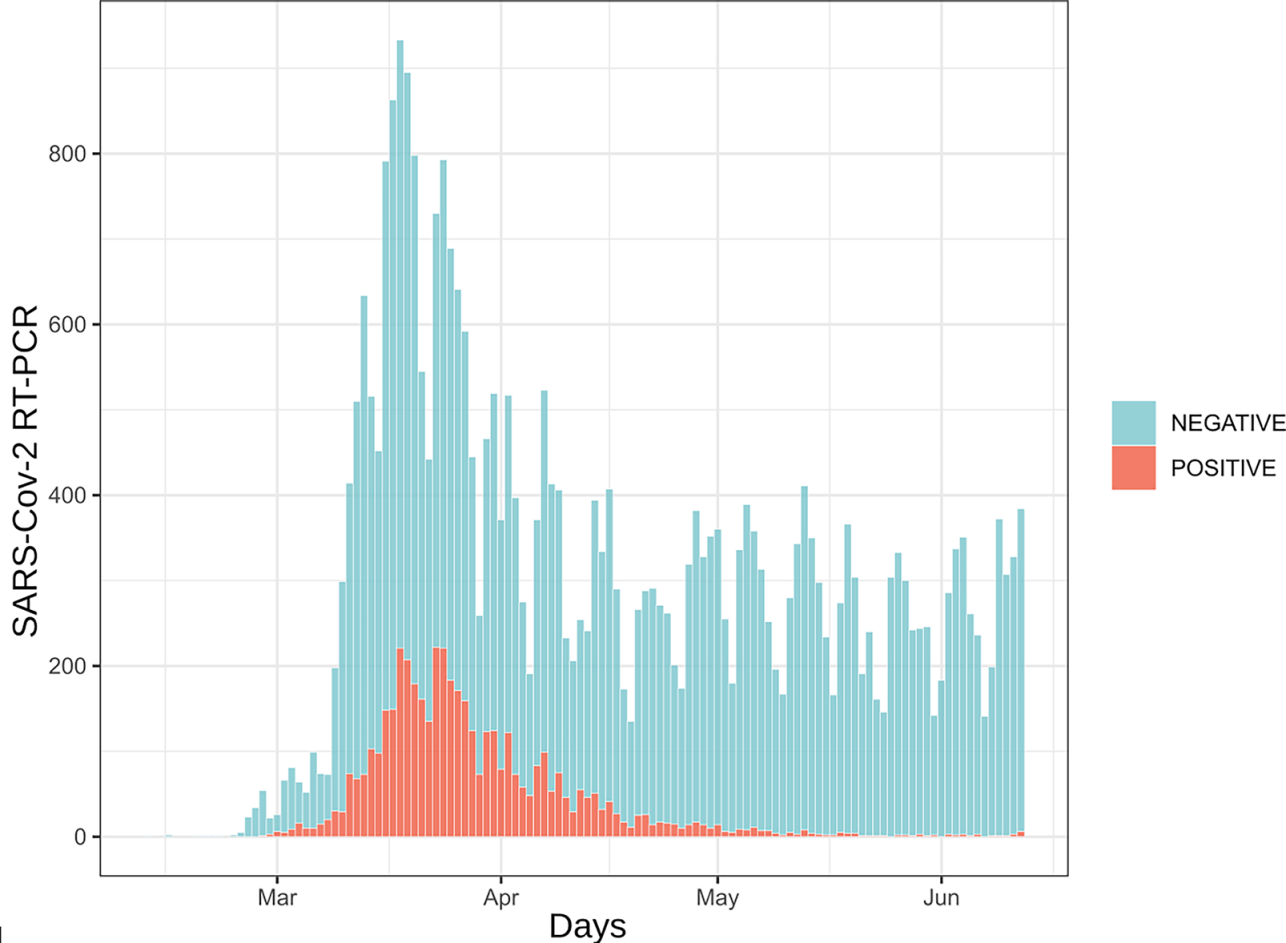
Daily number of SARS-CoV-2 RT-PCR assays. 35,137 analyzes are represented here. Samples are distributed according to their reception date. Blue bars represent samples for which RT-PCR results were negative (88%) while red bars depict positive samples (12%).

**Figure 2:**
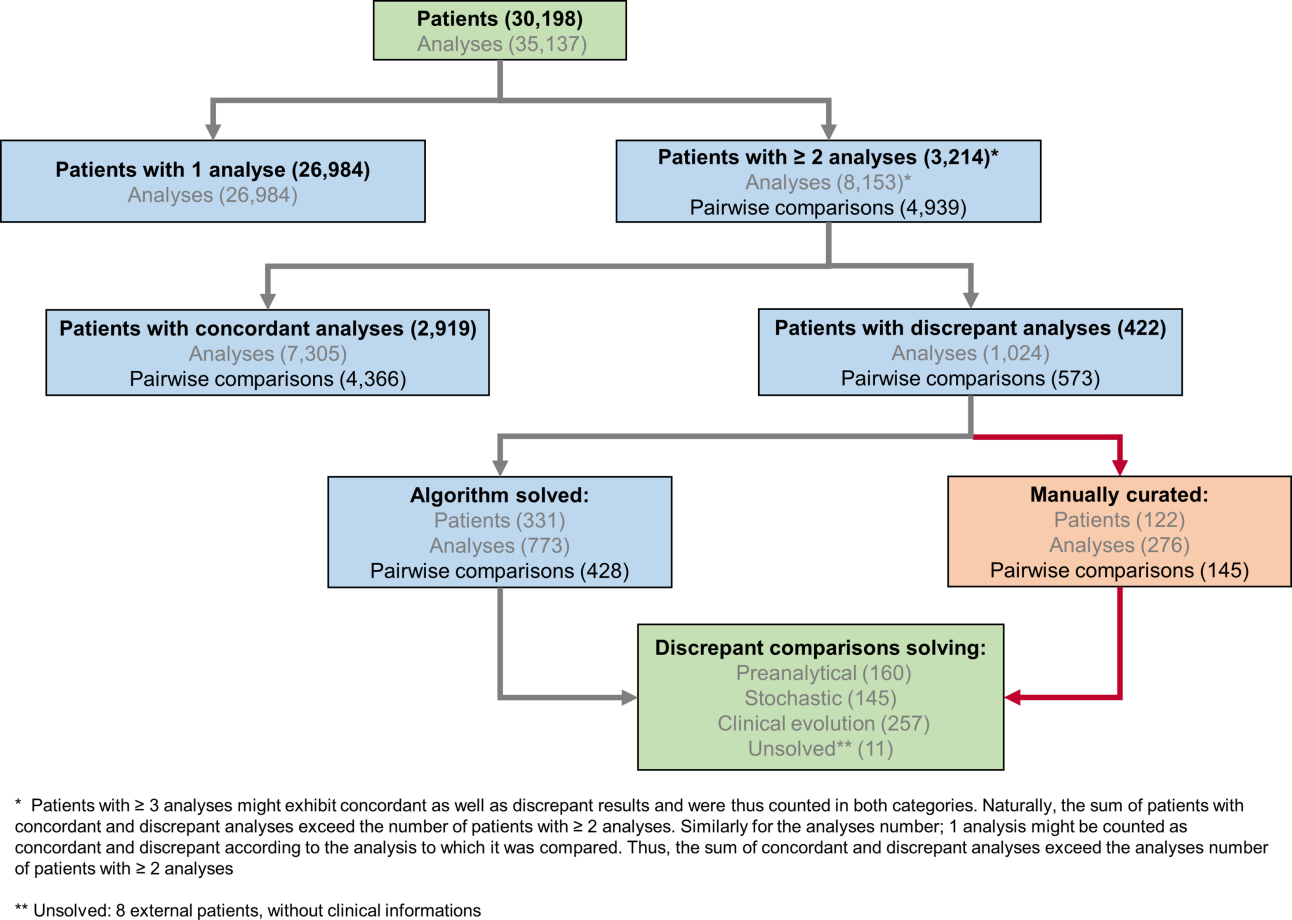
SARS-CoV-2 analytical flowchart. An R-based script was used to identify 3,214 patients with multiple, potentially discrepant, results (upper part of the chart) and therefore ensure their surveillance. Among these patients with multiple analyzes, 2,792 exhibited only concordant results, while 422 presented at least one discrepant pair of results. Of note, 127 patients had concordant as well as discrepant results; these latter were further processed by the algorithm as patients with discrepant results. When considering 4,939 pairwise comparisons of successive results for patients with multiple tests, 4,366 (88%) were concordant and 573 (12%) were discrepant. The algorithm processed discrepant results further and a potential explanation for the observed discrepancy was attributed either automatically or manually (lower part of the chart and **Supplementary Figure S1**).

Since the implementation of SARS-CoV-2 RT-PCR assays and for a period of four months, 30,198 patients were tested by RT-PCR at the Institute of Microbiology of the Lausanne University Hospital (CHUV). This corresponded to 35,137 samples, among which 4,545 (12.9%) returned a positive result, whereas 30,592 (87.1%) were negative. Upper respiratory tract (URT) samples represented 98% of the tested specimens (**Supplementary Table S1**). The peak of number of analyzes took place on March 18th with up to 1,007 analyzes processed during the same day (**Figure 1**). The developed algorithm allowed the laboratory to process 3,214 patients having at least two specimens, as detailed in the **Figure 2**.

### Algorithm-Based and Manual Biomedical Investigation of Discrepancies

**Figure 3:**
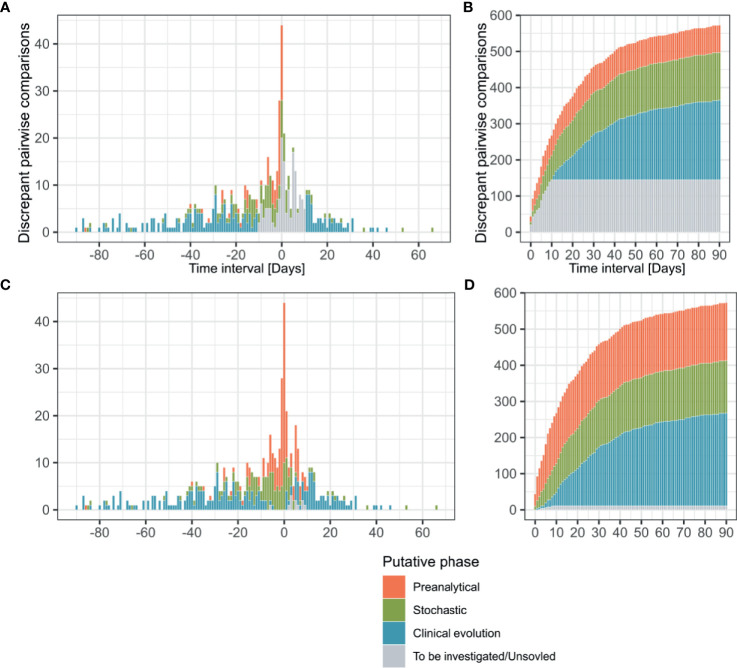
Time interval between discrepant analyzes, putative phase assignment and cumulative curves. Pairwise comparisons (n = 573) are represented according to the interval between their reception dates and colored depending on the analytical phase which best explained the observed discrepancy. Before manual curation discrepancies were classified by the algorithm as “Preanalytical”, “Stochastic”, “Clinical evolution” and “To be investigated” **(A, B)**. After manual curation comparisons previously categorized as “To be investigated” were reassigned to the same categories or as “Unsolved” **(C, D)**. Transitions from a negative to a positive result are represented in the positive side of x axe, while positive to negative transitions are plotted on the negative side **(A, C)**.

Our pipeline significantly reduced the number of discrepancies requiring human investigation and a probable explanation could be identified for most of the discrepant results. Indeed, 75% (n=428 of total 573) of the discrepant pairwise comparisons could be automatically attributed by the pipeline to a putative explanation, i.e., “stochastic”, “low yield” or “time delay” (**Figures 3A**, **B** and **Supplementary Table S3**). Only the remaining discrepancies (n=145) did not fit any of the solving rules encoded in the algorithm and required investigations based on the available analytical and clinical information (**Supplementary Table S4**).

The profiles of putative explanations for discrepancies evolved depending on the time interval between the compared analyzes (**Figure 3C**). In samples received during the same day, our assessment explained 77.3% (n=34/44) of the discrepancies as related to the preanalytical phase (i.e. explained by the sample type or collection in different health centers) and 22.7% (n=10/44) to stochasticity of the RT-PCR reaction (Ct value >35). The discrepancies in results for samples received 1-3 days apart were explained by factors affecting the preanalytical phase (55.4%, n=51/92), followed by stochasticity (32.6%, n=30/92). Interestingly, 7 of the discrepancies observed in the 1-3 days interval were explainable by nosocomial (n=6) or community (n=1) acquired infections based on health records, which explained the quick negative to positive transition. These 7 discrepancies were thus classified in the clinical evolution context. As for the 4 remaining discrepancies, clinical records were not available for 3 and the last one remained unexplained. Investigation of discrepancies between samples received 4-10 days apart again incriminated mainly the preanalytical phase (41.6%, n=55/132), followed by stochasticity (30.3%, n=40/132). As expected, the discrepancies imputable to the clinical evolution of the disease based on clinical records (new infection or infection resolution) was greater in the 4-10 days interval since it represented 22.7% of the discrepancies (n=30/132). The 7 remaining discrepancies in this time interval could not be explained, either in absence (n=5) or in presence (n=2) of clinical information. Over 10 day, the clinical evolution of the disease was the main explanation (72.1%, n=220/305) for discrepancies, as it was the default explanation retained by our automatic pipeline for discrepant results from samples collected more than 10 days apart in absence of any other explanation.

In the overall assessment of the 573 discrepancies from samples taken up to 90 days apart, 44.9% (n=257) of discrepancies could be explained by the clinical evolution of the disease (e.g. indications in clinical records for new contagion, time delay making new infection or infection resolution likely) (**Figure 3D**). 27.2% (n=160) of cases had arguments for factors incriminating the preanalytical phase (discrepant results among samples collected by different health centers, inclusion of samples rarely positive as blood); and 25.3% (n=145) of the discrepant comparisons could be explained by analytical stochasticity in presence of low RNA loads (Ct value > 35 for the positive sample followed or preceded by a negative sample). No clear explanation could be identified for 1.9% (n=11) of the discrepancies (classified as “Unsolved”). Among the unsolved situations, 8 samples were submitted to our laboratory by external care centers or private laboratories and clinical records were thus not accessible. No explanation for discrepancies could be found for 3 cases, despite the availability of full clinical documentation. Moreover, short-term negative to positive transitions were compatible with 21 nosocomial and 8 community-acquired infections based on clinical records (**Supplementary Table S4**).

### Evolution of Discrepancy Patterns Across the Epidemic Period

The pattern in transitions (negative result followed by a positive result or the reverse) among discrepancies evolved over the studied period of four months that correspond to the four first months of epidemic in our region. In the first two months (12.02-12.04.2020), 71.3% of observed transitions were negative to positive (n=154/216). Conversely, in the last two months (13.04-12.06.2020), 81.2% of the transitions went from a positive to a negative result (n=290/357), which contributed to an overall trend of 61.4% of positive to negative discrepancies (n = 352/573) (**Figures 3A** **, C**). Such observation was expected and imputable to follow-up of patients with resolving infections.

### Sustained RT-PCR Positive Results in Patients

**Figure 4:**
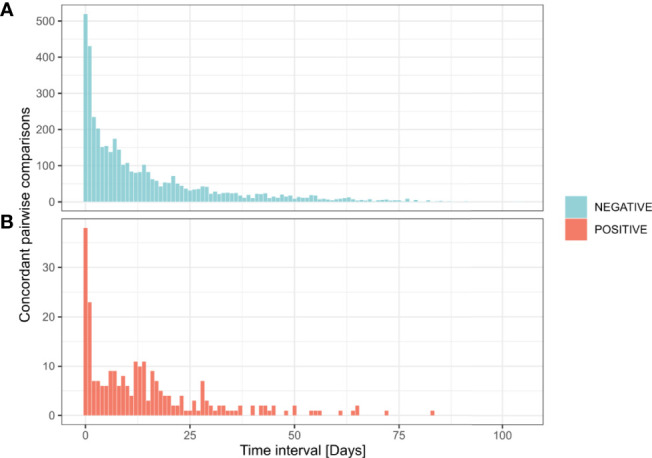
Time interval between successive concordant analyzes from the same patient. Distribution of 4,366 pairwise concordant comparisons according to time interval between their reception dates. Negative concordant comparisons for SARS-CoV-2 detection (n = 4,116) **(A)**. Positive concordant comparisons for SARS-CoV-2 detection (n = 250) **(B)**.

**Figure 5:**
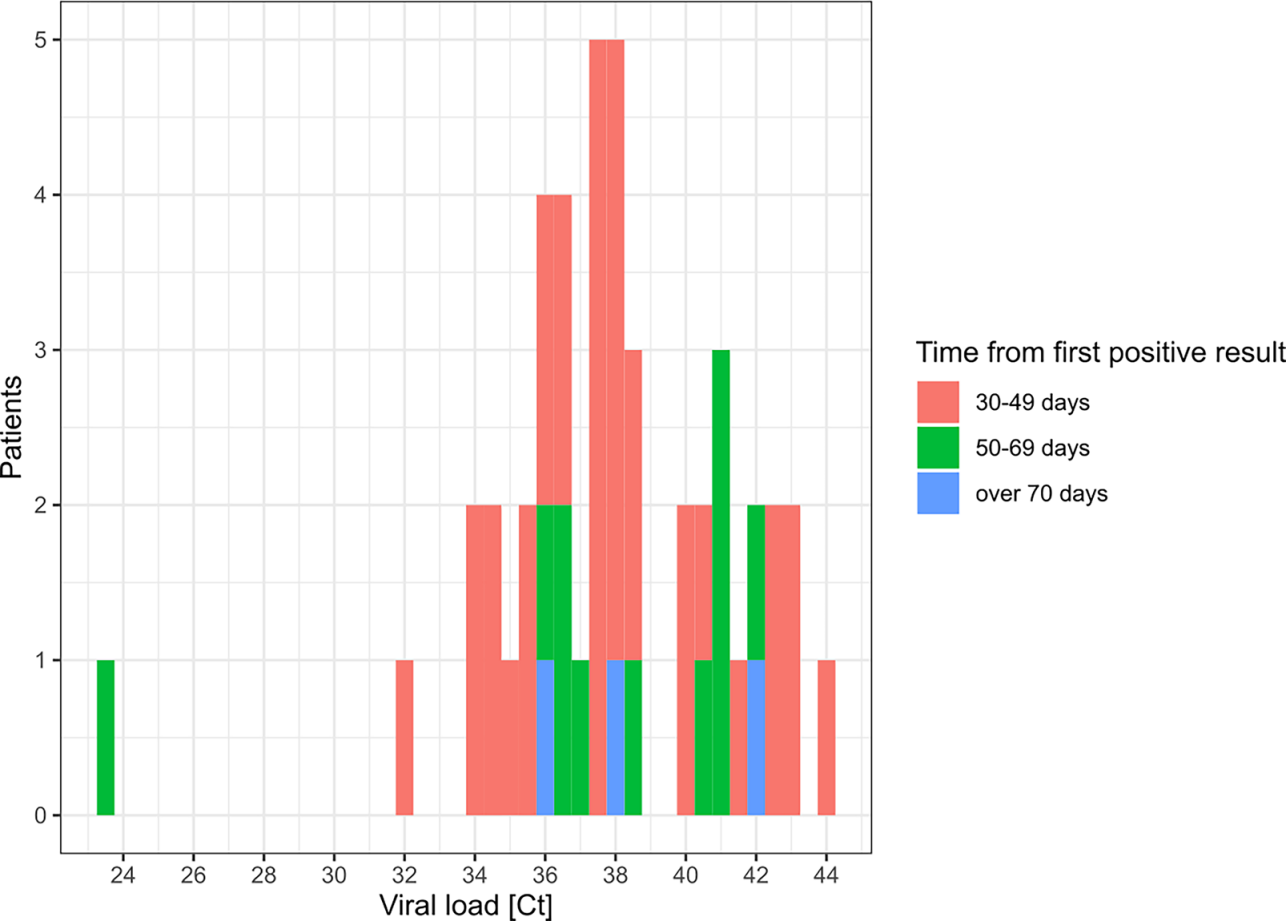
Distribution of Ct values for patients with sustained RT-PCR positive results. This histogram represents the Ct value (maximal if more than 1) for SARS-CoV-2 detection by RT-PCR in the last positive sample of patients with sustained positivity in samples taken 30 days apart or more.

Besides discrepancies, we also investigated sustained positivity in patients. In our analysis, the longest time interval between two positive results from the same patient was of 83 days (**Figures 4A, B** and **5**). Considering the time interval between their first and the last positive result, we observed 32, 11, and 3 patients with sustained positivity in samples taken over 30, 50 and 70 days apart, respectively. This observation questions the presence of active viral replication or only of viral traces remaining from the resolving infection. In line, the last sample of these long-time carriers displayed a low viral load with Ct values around 35 (corresponding to 3,800 copies/ml) in all but one notable exception (**Figure 5**).

### Pairwise Analyzes Highlight The Consistency of SARS-CoV-2 RT-PCR Results

Reports questioned the performance of RT-PCR for SARS-CoV-2 detection, which supported recommendations for repeated testing (13). In our dataset, only 2.5% of the negative results obtained from an URT sample (n=733/29,714) were followed by an additional analysis 1 hour to 3 days after initial testing. In comparison, 0.6% (n=28/4,451) of positive results from URT samples were followed by a second analysis over the same time interval. Thus, if repeated testing remained limited, a negative result was still significantly more often challenged than a positive result by clinicians (Pearson’s Chi-squared Test, p < 0.001, OR = 4.0).

An evaluation of the performance of the RT-PCR for SARS-CoV-2 detection based on samples collected over a short time interval showed a good level of concordance. Indeed, an initial negative result in an URT sample was confirmed in 99.2% (n=243/245) of cases for patients tested twice on the same day (**Table 1**); both discrepancies could be explained by stochasticity since associated to high Ct values. Conversely, a first positive result was confirmed in 88.9% (n=24/27) of cases; two discrepancies could be explained by stochasticity too, while the third involved a positive nasopharyngeal swab and a negative throat swab, a sample site shown to be less sensitive for SARS-CoV-2 detection (14). As expected, concordance rates diminished over time: negative result concordance for URT samples went from 99.2% for samples collected along the same day to 94.2% for samples collected 1-3 days apart. On the same time intervals, the concordance for positive results evolved from 88.9% to 70.0%.

**Table 1:** Agreement between URT samples collected in a repeatedly in patients over a short time-interval.

	Same day(n = 272)	1-3 day(n = 826)	4-6 days(n = 489)	7-10 days(n = 585)
Concordant Negative results	243	750	430	516
Concordant Positive results	24	21	7	20
Negative to Positive transition	2	46	38	28
Positive to Negative transition	3	9	14	21
**Concordant negative agreement**	99.2%	94.2%	91.9%	94.9%
**Concordant positive agreement**	88.9%	70%	33.3%	48.8%

## Discussion

This work presents the importance of a homemade algorithm developed in response to the need for quality surveillance of SARS-CoV-2 RT-PCRs which throughput exceeded the ability to conduct manual biomedical validation for each sample. Applied to the 35,137 SARS-CoV-2 RT-PCRs performed in our diagnostic laboratory from February 12 to June 12, 2020, the algorithm identified 3,214 patients owing multiple tests. These patients represented an opportunity for quality assessment of our analyzes, but also required careful attention to investigate potential discrepancies. Among the 3,124 patients tested multiple times, we observed a majority (86.8%) of concordant results, mostly negative (96.8%). Of these patients, 422 exhibited at least one pair of discrepant results. Together, the clinical evolution of the disease (44.9%), preanalytical factors (27.9%) and stochasticity around the limit of detection (25.3%) were the most likely explanations retained for the 573 observed discrepancies. Only 1.4% of the cases remained unexplained because clinical records were not available. Despite availability of all records, 0.5% of the results remained unexplained.

Expectedly, the natural evolution of the disease explained most of the observed discrepancies. The preanalytical factors were the second most frequent source of discrepancies. This observation is in line with previous reports that described this testing phase as an important source of errors in general in clinical laboratories (Plebani, 2006; West et al., 2017). Stochasticity was the only identified source of discrepancy directly related to the analytical phase. However, the clinical impact of these discrepancies could be limited since low viral loads are expected in the late course of the disease, at time when the infectivity might be diminished (Jacot et al., 2020; Moraz et al., 2020; Yu et al., 2020). Of note, samples were analyzed according to the laboratory workflow. Indeed, samples belonging to the same patient were not systematically analyzed with the same method. Thus, the difference in LOD proper to each assay might also be a source of the discrepancies classified as stochastic (Opota et al., 2020).

Sustained positivity with high Ct values was observed in 45 patients (Fig. 5). According to studies focusing on prolonged presence of viral nucleid acid, viral traces might not be associated with effective infectiousness and mostly correspond to nonculturable samples (Hong et al., 2020; Huang et al., 2020; Wolfel et al., 2020). Nevertheless, many factors can impact the viral load in a clinical specimen (i.e. quality of the sampling); therefore, several co-variables have to be taken into consideration to address the contagiousness (Jacot et al., 2020; Moraz et al., 2020).

Our results support the good performance of RT-PCR in URT samples for SARS-CoV-2 detection. Nevertheless, these agreement rates should be considered with caution, particularly due to the small number of positive samples for which additional testing was requested. Another limitation of our work is that while we intended to use an unbiased algorithm stable over time to investigate discrepancies in results, some of the applied criteria were partly arbitrary (e.g. the 10 days limit to consider discrepancies as due to the clinical evolution of the disease). Furthermore, our process retained a single explanation for each observed discrepancy, while more could be applicable. While arguable, these choices were made to fit a strategy of quality monitoring. Indeed, the primary aim of the present methodology was to attribute the observed discrepancies to the most likely explanation to focus on truly unexplainable and clinically problematic cases. Clinical laboratory vulnerabilities during the COVID-19 pandemic were the subject of a recent publication by Lippi et al. (Lippi and Plebani, 2020). Our assessment overlaps with some of the preanalytical culprits identified by the authors such as specimen collection (see “detailed explanations” **Supplementary Tables S3** and **S4**). However, some other potential vulnerabilities were not considered as probable causes for discrepancies in our assessment, since they are covered by other pre-existing quality management procedures in our laboratory. For instance, samples missing patient identification were systematically rejected. Moreover, internal extraction controls and amplification controls were systematically included to detect samples that might contain interfering substances compromising the amplification (Poljak et al., 2020).

This is the first implementation in the clinical microbiology facility of the Lausanne University Hospital of a quality monitoring tool resembling a “Delta check” applied in clinical chemistry laboratories (Schifman et al., 2017). “Delta checks” are usually restricted to analytes exhibiting limited short-term variations and is as such unsuitable to microbiology results. Yet, helped by an algorithm capable of considering expected variations in results, we could adapt the concept of “Delta check” to SARS-CoV-2 RT-PCR. Similar longitudinal observation of results and algorithm-based selection of “cases to investigate” could also be applied to other high-throughput microbiology laboratory assays, either by the implementation of an ad-hoc software as presented here or by rules embedded in the LIS.

The strategy applied for the management of large amount of SARS-CoV-2 samples in our center, comprising the extension of our analysis platform set and the introduction of an automatic validation, allowed to reduce the median TAT from 6.9h to 4.8h between February 24^th^ and June 9^th^, 2020 (Marquis et al., 2021).

However, the duration of the biomedical validation step, depending on the pathogen and the epidemiological situation, remains to be assessed.

This work emphasized the benefit of an automated algorithm capable of finding discrepant results and attributing them to corresponding testing phases. This computer-aided methodology outlines that besides the expected evolution of the disease, most of discrepant results are compatible with preanalytical factors. Moreover, most of URT samples collected repeatedly in a short timeframe showed consistent results, displaying the good reproducibility of the RT-PCR for SARS-CoV-2 detection. Application of this method for quality monitoring enabled to focus on problematic cases requiring biomedical expertise while maintaining an acceptable TAT.

## Data Availability Statement

The code of the algorithm presented in this article can be found under https://github.com/valscherz/SARS-CoV-2_discrepant_screen.

## Ethics Statement

Ethical review and approval was not required for the study on human participants in accordance with the local legislation and institutional requirements. Written informed consent from the participants’ legal guardian/next of kin was not required to participate in this study in accordance with the national legislation and the institutional requirements.

## Author Contributions

VS and LM conducted the analyses under GG, KJ, and OO supervision for study design and data interpretation. VS designed the R script. LM investigated discrepant cases. LM and VS wrote the original manuscript. All authors contributed to the article and approved the submitted version.

## Conflict of Interest

The authors declare that the research was conducted in the absence of any commercial or financial relationships that could be construed as a potential conflict of interest.

## Publisher’s Note

All claims expressed in this article are solely those of the authors and do not necessarily represent those of their affiliated organizations, or those of the publisher, the editors and the reviewers. Any product that may be evaluated in this article, or claim that may be made by its manufacturer, is not guaranteed or endorsed by the publisher.
