# Supplementary material for: Computer-Aided Medical Microbiology Monitoring Tool: A Strategy to Adapt to the SARS-CoV-2 Epidemic and That Highlights RT-PCR Consistency
Source: Front Cell Infect Microbiol. 2021 Sep 13;11:594577. doi: 10.3389/fcimb.2021.594577 (PMC8475725; doi:10.3389/fcimb.2021.594577)
Supplement: Supplementary file 1 [file DataSheet_1.docx]

Supplementary Figure S1. Decisional algorithm flowchart.

This flowchart represents the decisional algorithm coded in R to compare results and to identify cases requiring further investigation. This algorithm was applied to a spreadsheet table extracted from the Laboratory Informatic System (LIS). Analysis of each patient, ordered based on reception time, were processed according to this workflow.


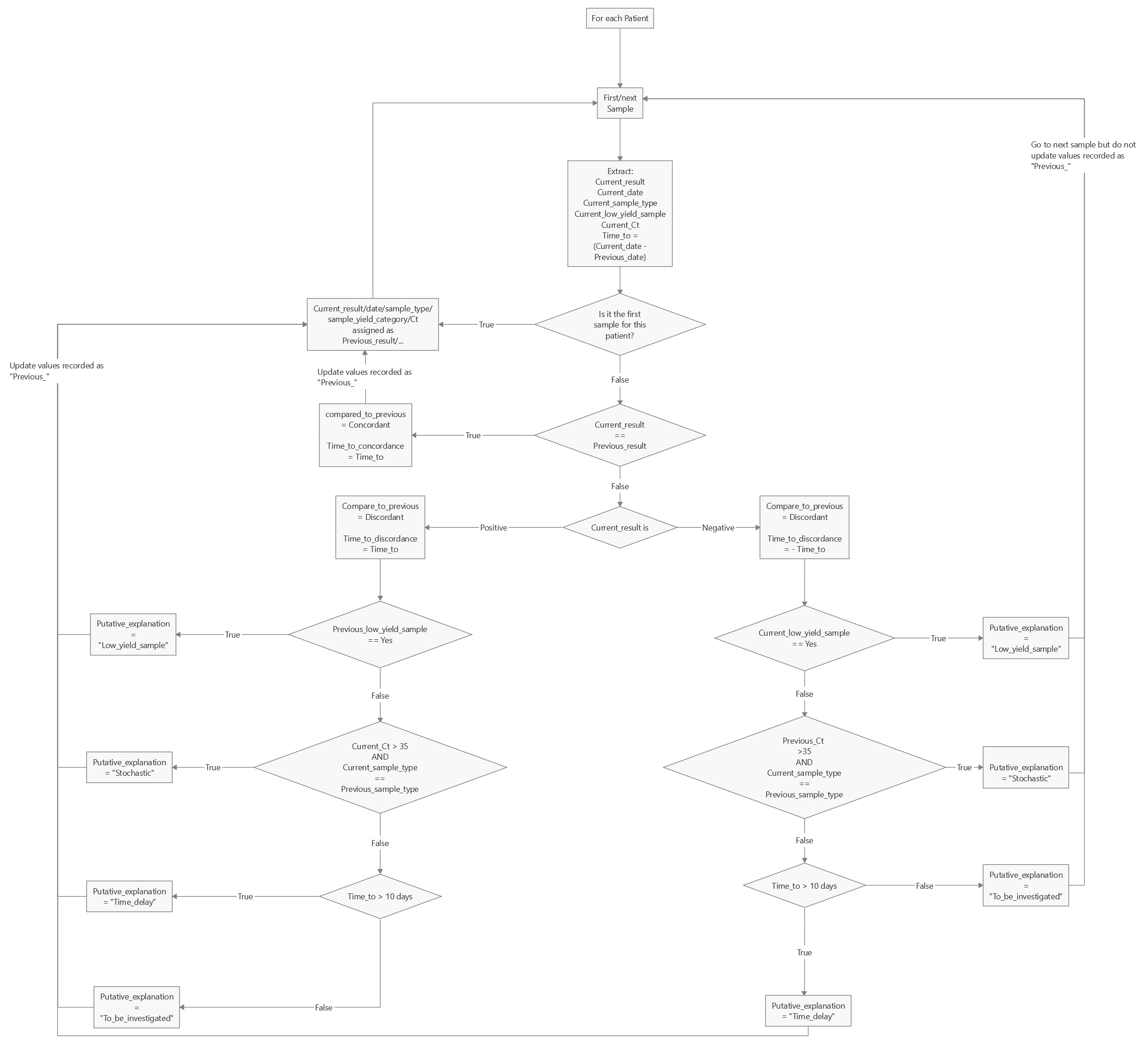


As for in R language, “==” stands for “equal to?” and “=” stands for the assignment of value to variable.

Supplementary Table S1. Results per sample type categories

| Sample type | Negative | Positive |
| --- | --- | --- |
| Blood* | 50 (98%) | 1 (2%) |
| CSF* | 45 (100%) | 0 (0%) |
| LRT samples | 243 (84.4%) | 45 (15.6%) |
| Rectal swab | 227 (93%) | 17 (7%) |
| Urine* | 7 (100%) | 0 (0%) |
| URT samples | 29928 (87%) | 4478 (13%) |
| Other* | 92 (95.8%) | 4 (4.2%) |
| Total | **30592** | **4545** |

CSF: Cerebrospinal fluid

Lower respiratory tract (LRT): Bronchoalveolar lavage, sputum, endotracheal secretion, bronchial aspiration.
Upper respiratory tract (URT): Nose swab, nasopharyngeal secretion, throat swab, nose and throat swab, mouth swab, oropharyngeal swab.

Other: vaginal swab, fetal samples, placenta swab, amniotic fluid, ear swab, skin swab, kidney biopsy, pericardial fluid, pleural effusion.

*These samples exhibited a low yield (<5% of positivity) and were thus classified as “low yield samples”

Supplementary Table S2. Algorithm criteria: examples of classification of putative

| **Patient** | **Sample** | **Sample type** | **Result** | **Ct** | **ΔTime** | **Classification** | **Explanation** | **Pairwise comparison** |
| --- | --- | --- | --- | --- | --- | --- | --- | --- |
| **A** | 1 | Nasopharyngeal swab | POS | <35 |  |  | Sample type rarely or never observed as positive (i.e. less than 5% of times, Supplementary Table S1) |  |
| **A** | 2 | Blood | NEG |  | <10 D | Low yield |  | 1 Discordant |
| **A** | 3 | Nasopharyngeal swab | POS | <35 | <10 D |  |  | 1 Concordant  1 Discordant |
| **B** | 1 | Nasopharyngeal swab | NEG |  |  |  | Ct values over 35; close to the analytic limit of detection (a Ct of 35 corresponds to 3,800 copies/ml) |  |
| **B** | 2 | Nasopharyngeal swab | POS | >35 | > or <  10 D | Stochastic |  | 1 Discordant |
| **C** | 1 | Nasopharyngeal swab | POS | <35 |  |  | Time interval between the two discrepant samples is over 10 days. The discrepancy could be explained by the evolution of the disease (new infection or disease resolution) |  |
| **C** | 2 | Nasopharyngeal swab | NEG |  | >10 D | Time delay |  | 1 Discordant |
| **D** | 1 | Nasopharyngeal swab | NEG |  |  |  | None of the previous criteria are met. Further investigation by medical microbiologist is requested |  |
| **D** | 2 | Nasopharyngeal swab | POS | <35 | <10 D | To be investigated |  | 1 Discordant |
|  |  |  |  |  |  |  |  |  |
|  |  |  |  |  |  |  |  |  |

Supplementary Table S3. Output of automated discrepancy solving

| Detailed explanation | Classification | Category* | Processed | n |
| --- | --- | --- | --- | --- |
| Time laps >10 days, new infection likely | Time delay | Clinical evolution | Algorithm | 63 |
| Time laps >10 days, solved infection likely | Time delay | Clinical evolution | Algorithm | 157 |
| Samples rarely or never positive | Low yield | Preanalytical | Algorithm | 76 |
| Ct>35 limit of the detection, subjected to stochasticity | Stochastic | Stochastic | Algorithm | 132 |
| None of previous explanation, to manually curate | To be investigated | To be investigated | Algorithm | 145 |
| Total |  |  |  | **573** |

*Classification provided by the R script were grouped into these categories in figures.

Supplementary Table S4. Counts of discrepancies after manual investigation

| Detailed explanation | Classification | Category* | Processed | n |
| --- | --- | --- | --- | --- |
| Time laps <10 days, community-acquired context | Clinical context | Clinical evolution | Manually | 8 |
| Time laps <10 days, nosocomial context or hospitalized >48h | Clinical context | Clinical evolution | Manually | 21 |
| Time laps <10 days, resolved infection likely | Clinical context | Clinical evolution | Manually | 8 |
| Samples collected in different settings (different department/ hospitals/ clinic/ etc.) | Sample quality | Preanalytical | Manually | 44 |
| Two samples of different nature (i.e. anal swab VS oropharyngeal swab) | Different sample type | Preanalytical | Manually | 40 |
| Ct close to 35, limit of the detection, subjected to stochasticity | Stochastic | Stochastic | Manually | 13 |
| Extern case with no clinical information available | Extern clinical information NA | Unsolved | Manually | 8 |
| Intern case with no explanation for the observed discrepancy/ no clinical information | Intern clinical information NA | Unsolved | Manually | 3 |
| Total |  |  |  | **145** |

*Classification provided by the R script were grouped into these categories in figures.

NA: Not Available
